# Evaluation of Marine Microorganism-Derived Oils Containing EPA and DHA for Triglyceride Improvement and Blood Circulation Enhancement

**DOI:** 10.4014/jmb.2412.12009

**Published:** 2025-04-09

**Authors:** Tae Yeon Yin, Jung-rae Rho, Yeong Du Yoo, Eun Ju Jeong, Jaeyeon Park, Eun Young Yoon

**Affiliations:** 1 Advanced Institute of Convergence Technology, Suwon 16229, Republic of Korea; 2 Department of Oceanography, Kunsan National University, Kunsan 54150, Republic of Korea; 3 Department of Green Bio Science, Gyeongsang National University, Jinju 52725, Republic of Korea

**Keywords:** Protist, *Oxyrrhis*, functional food material, biomarker, triglyceride improvement, blood circulation

## Abstract

Cardiovascular diseases (CVDs) present significant health challenges globally, with dysregulated triglyceride levels and impaired endothelial function being key contributors to their pathogenesis. In this study, we explore the potential of marine Microorganism-derived oils rich in eicosapentaenoic acid (EPA) and docosahexaenoic acid (DHA) in addressing these physiological phenomena associated with CVDs. Exploring marine resources for physiologically active compounds intertwines with ecological considerations, highlighting the interconnectedness between human health and the environment. Marine microorganisms, particularly protist *Oxyrrhis*, are abundant sources of bioactive compounds, including EPA, known for their lipid-lowering effects and vascular benefits. Additionally, marine biodiversity offers a rich reservoir of compounds with potential health-promoting properties. In this study, we evaluated the efficacy of EPA- and DHA-containing oils in improving triglyceride levels and enhancing blood circulation. Lipoprotein lipase (LPL) and nitric oxide (NO) were identified as pivotal players in the mechanism of action, with LPL facilitating neutral fat metabolism and NO regulating vascular tone and function. This study contributes to the growing evidence supporting the use of marine-derived compounds in preventing and managing CVD, emphasizing the ecological importance of marine ecosystems to human health by leveraging the bioactive potential of marine biodiversity sustainably.

## Introduction

Cardiovascular diseases (CVDs) have a complex pathology and are defined by vascular function and elevated triglyceride levels. Dysfunctional endothelium-dependent relaxation leads to a chronic and abnormal elevation of vascular resistance, resulting in hypertension [1]. Elevated triglyceride levels are independently associated with an increased risk of cardiovascular disease [2], making proper triglyceride levels, endothelial function, and vascular relaxation critical physiological factors in the prevention of CVDs. A crucial treatment modality for CVDs involves the induction of vasorelaxation to reduce hypertension and improve triglycerides in the blood [3]. Therefore, interventions aimed at enhancing vasorelaxation and controlling triglyceride levels are crucial for mitigating the risk and progression of CVDs. Various natural substances and bioactive compounds can be utilized to achieve such physiological goals, with marine resources being a particularly noteworthy area of interest.

In recent years, the exploration of marine bioresources for physiologically active compounds has garnered increasing interest in the development of new drugs and health foods [3]. Marine microorganisms have emerged as an abundant source of antioxidants [4]. They contain various physiologically active substances that lower cholesterol, reduce blood pressure, promote healthy digestion, and exhibit antioxidant activities [5]. Among marine microorganisms, *Nannochloropsis* stands out because of its rich content of soluble and insoluble polysaccharides, proteins, and eicosapentaenoic acid (EPA). EPA and docosahexaenoic acid (DHA) effectively regulate blood lipid levels, which are critical in managing and preventing CVDs [6]. Since 2003, interest in EPA and DHA derived from *Odontella* and *Ulkenia* has been growing due to their anti-inflammatory properties and potential for treating brain and heart diseases [7, 8]. Furthermore, A previous study of *Oxyrrhis* lipid extract revealed a lipid-rich composition, including omega-3 fatty acids such as EPA and DHA, and demonstrated its efficacy and benefits in lowering blood pressure and improving triglyceride levels [9].

In this study, (LPL) and (NO) were selected as biomarkers because they are directly involved in lipid metabolism and vascular function, which are important in the pathophysiology of CVD. LPL plays a significant role in hydrolyzing triglyceride-rich lipoproteins, influencing lipid clearance, and modulating lipid levels, whereas NO is a key regulator of vascular tone and blood flow. Assessing these biomarkers allows for a comprehensive understanding of how *O. marina* supplementation may influence lipid metabolism and endothelial function.

Stimulation by apolipoprotein C-II (ApoCII), found in chylomicrons and very low-density lipoproteins (VLDL), catalyzes the breakdown of neutral fat into free fatty acids and glycerol [10]. LPL activation varies between adipose and muscle tissues, increasing postprandially in the adipose tissue and during fasting in the muscles [11]. Although normally present in minimal amounts in the blood, intravenous administration of heparin prompts the release of LPL attached to vascular endothelial cells, subsequently increasing its activity [12]. Elevated LPL activity is correlated with heightened decomposition of neutral fatty acids, thereby potentially improving blood triglyceride levels [13].

NO is generated within endothelial cells by the endothelial isoform of nitric oxide synthase (eNOS) from the amino acid L-arginine, and it plays a pivotal role in maintaining vascular tone and regulating various vascular functions, including growth, thrombogenesis, lipid metabolism, and inflammation [14]. Endothelial NO primarily mediates vascular expansion and modulates leukocyte and endothelial cell adhesion while suppressing the secretion of vascular contracting peptides [15]. As a potent vascular expander, the effects of NO on vascular expansion can be assessed by measuring the concentrations of NO and its metabolites in blood or urine [16, 17].

In this study, we aimed to evaluate the effects of oil containing EPA and DHA, derived from the marine heterotrophic dinoflagellate *Oxyrrhis marina*, which has high contents of bioavailable omega-3 polyunsaturated fatty acids, particularly EPA and DHA. These compounds are critical in modulating inflammation and lipid metabolism, thus enhancing their potential effects on CVD by improving blood circulation. To assess the role of *Oxyrrhis* extracts in promoting cardiovascular health, we examined the oil’s ability to enhance NO production, which facilitates vasodilation, and to increase LPL expression, which aids in the breakdown of triglycerides.

## Materials and Methods

### Preparation of *O. marina* Extracts and Fractions

**Fig. 1:**
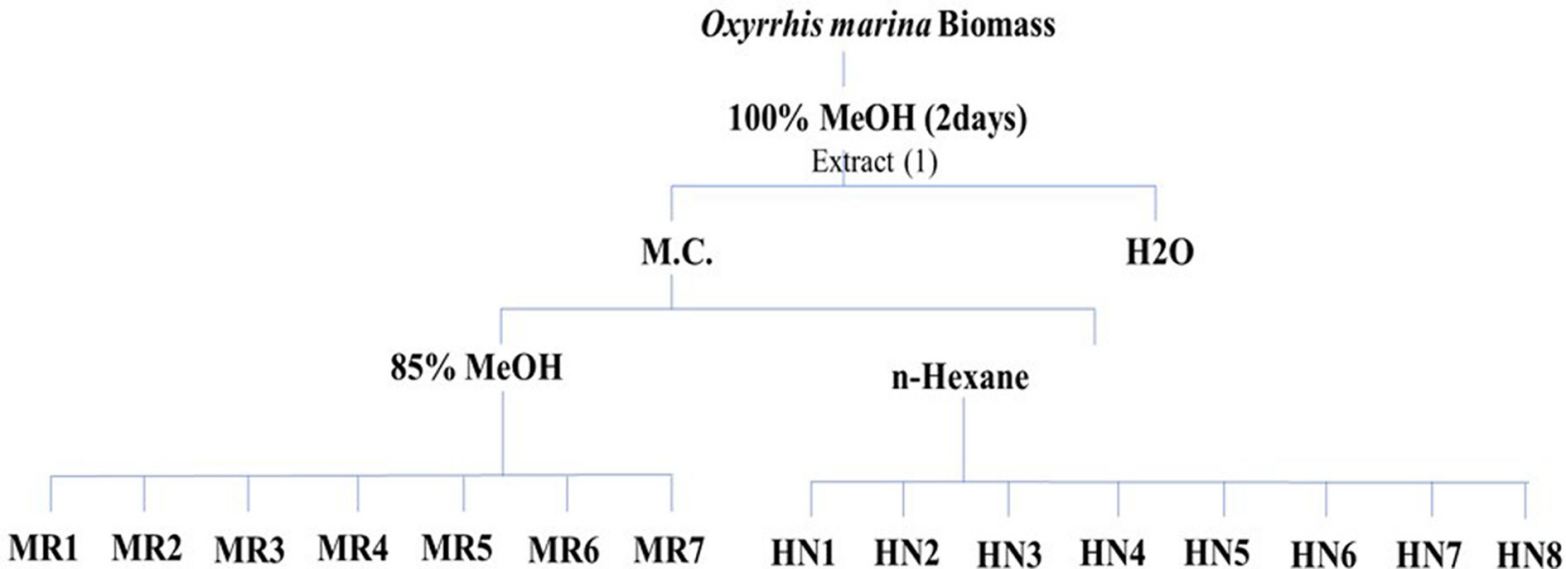
Extraction and fractionation of *Oxyrrhis marina* using flash chromatography.

*Oxyrrhis marina* (500 L) extract was obtained using 100% methanol for 48 h at 20-22°C. The extracts were centrifuged at 3,000 rpm for 10 min, and the supernatant was filtered and concentrated using a rotary evaporator (Eyela N-1100; Tokyo Rikakikai Co. Ltd., Japan). The organic fraction was first partitioned with water and methylene chloride to test the activity of the extract. Subsequently, the methylene chloride fraction collected from the lower phase of the separation funnel was transferred, and the mixture was divided into 85% methanol and *n*-hexane layers by adding a mixed solution and hexane mixed with 85% methanol and 15% water. Samples were prepared using reversed-phase silica flash chromatography for the 85% methanol layer, and specific fractions were collected sequentially. Silica flash chromatography was used for the *n*-hexane layer, and the fractions were collected according to predefined solvent gradients ranging from low to high polarity (Fig. 1).

### Cell Culture and Treatments

The human hepatocarcinoma cell line HepG2 used in this experiment was obtained from the Korea Cell Line Bank (KCLB No. 88065). Dulbecco’s modified Eagle’s medium (DMEM) supplemented with 10% fetal bovine serum (FBS) (Gibco, USA), 100 units/ml penicillin (Invitrogen, USA), and 100 μg/ml streptomycin was used as the cell culture medium. The cells were cultured at 37°C in a humidified incubator containing 5% CO_2_. Human umbilical vein endothelial cells (HUVECs) were purchased from Thermo Fisher Scientific (USA) and were cultured in endothelial cell basal medium-2 (EBM-2; Lonza, USA) supplemented with EGM-2 SingleQuots^®^ supplement (Lonza). The cells were incubated at 37°C with 5% CO_2_ saturation.

### Cell Viability Assay in HepG2 Cells and HUVECs

Cell viability was determined using a colorimetric MTT assay based on the reduction of MTT (Sigma-Aldrich) to formazan by cellular dehydrogenase. Cells were seeded into 96-well plates (1 × 10^4^ cells/well). The cells were cultured for 24 h at 37°C under a humidified atmosphere containing 5% CO_2_. HepG2 cells and HUVECs were treated with various concentrations (1-20 μg/ml in PBS) of *O. marina* extract and cultured for 24 h and 48 h, respectively. MTT stock solution was added to each well (2 mg/ml), and the cells were incubated under the same conditions for another 2 h. Cell viability was measured by analyzing absorbance at 450 nm using an ELISA reader and comparing the results with those of the control group.

### Western Blot Assay to Assess LPL Expression in HepG2 Cells

Western blotting was performed to analyze lipase levels in HepG2 cells. A sample to be tested on HepG2 cells was treated at a concentration of 20 μg/ml and cultured for 24 h. Next, the cells were collected using a lysis buffer (M-PER Mammalian Protein Extraction Reagent 78501) containing a protease inhibitor cocktail (Thermo Fisher Scientific, Prod #78425) and centrifuged at 12,000 rpm for 20 min to obtain the supernatant. The protein levels in the collected supernatant were quantified using bovine serum albumin (BSA). A total of 30 μg of lysate was placed in 5X sample buffer and inactivated at 100°C for 5 min, followed by 10% sodium dodecyl-sulfate polyacrylamide mini-gel electrophoresis (SDS-PAGE). The separated proteins were transferred onto a polyvinylidene difluoride (PVDF) membrane, and 5% skim milk was poured into the membrane to prevent non-specific binding of the antibodies. The membrane was then submerged and incubated at room temperature for 1 h. Anti-LPL (Santa Cruz, sc-3759 HRP) and anti-β-actin antibodies (Cell Signaling Technology, USA) were diluted in 1% skim milk, followed by overnight incubation at 4°C. They were then washed with TBS-T for 10 min thrice. Subsequently, the secondary antibody was incubated for 1 h at room temperature after anti-rabbit IgG and anti-mouse IgG were diluted with horseradish peroxidase (HRP) in 1% skim milk. Then, the membrane was washed thrice for 10 min with TBS-T and reacted with ECL western blotting detection agent (Amersham), followed by evaluation using a ChemiDoc image analyzer (Bio-Rad, USA).

### Quantification of NO in HUVECs

HUVECs were dispensed into 96-well plates (1 × 10^4^ cells/well) and incubated for 24 h at 37°C in a 5% CO_2_ incubator, followed by treatment with *Oxyrrhis* extracts at various concentrations (1-20 μg/ml in PBS) and incubation for 48 h. Nitrite concentration in the culture medium was measured using the Griess reagent (Promega, USA). The same amount of Griess reagent was mixed with 100 μl of the culture medium and reacted at room temperature in the dark for 20 min. The absorbance was measured at 540 nm using an ELISA reader. The amount of NO secreted was measured and compared with that in the control group.

### Statistical Analysis

The results of this experiment were analyzed using the statistical analysis software SPSS version 18.0, and the mean and standard deviation were calculated. An independent *t*-test was performed to verify significance at *p* < 0.05 to compare means between the two groups. One-way ANOVA was conducted to compare means among three or more groups, and Duncan’s multiple comparison test (*p* < 0.05) was used to verify the significance of the means.

## Results

### Improvements in Neutral Fat by *Oxyrrhis* Fractions

**Fig. 2:**
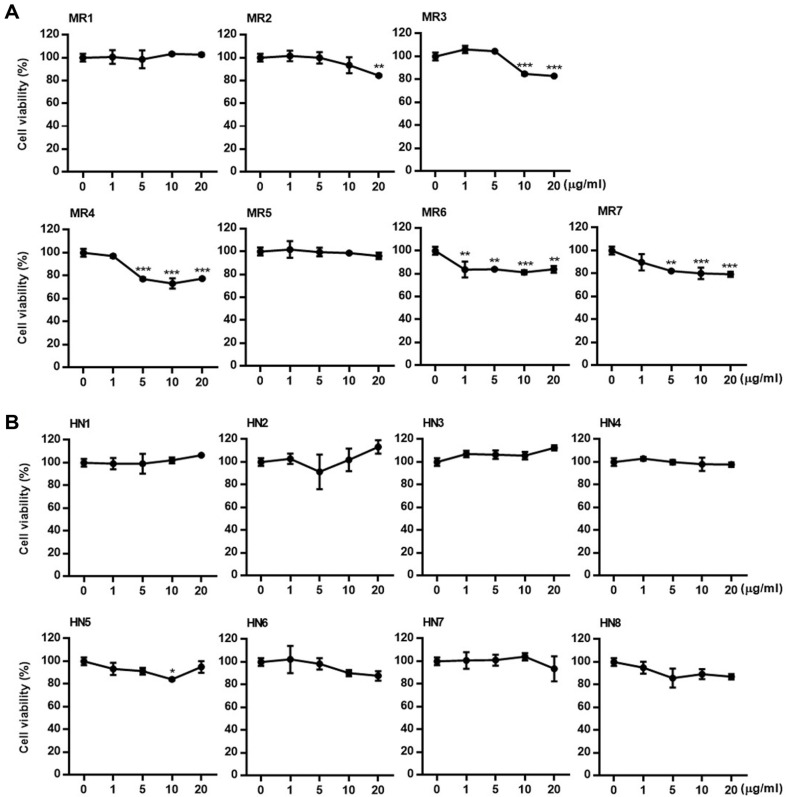
Cytotoxicity of subfractions of *Oxyrrhis marina* extract against HepG2 cells. HepG2 cells were treated with subfractions MR1–7 of the 85% methanol fraction and subfractions HN1–8 of the *n*-hexane fraction of *O. marina* extract. After 24 h incubation, cell viability was measured using the MTT assay. Results are presented as the mean ± SD of triplicate experiments; **p < 0.01 and ***p < 0.001 compared to non-treated cells.

**Cytotoxicity of polar fractions of *O. marina*.** Before the evaluation of the LPL regulatory activity of the extract and fractions of *O. marina*, the cytotoxicity of the samples was evaluated using the MTT assay in HepG2 cells. From the *O. marina* extract, two main fractions-the 85% methanol fraction and the *n*-hexane fraction—were obtained, and 15 subfractions were prepared from these fractions. The cytotoxicity of each subfraction was systematically evaluated. As shown in Fig. 2, HepG2 cells were treated with each subfraction at concentrations of 1, 5, 10, and 20 μg/ml, and an MTT assay was performed 24 h later. Cells treated with subfractions HN1–8 of the *n*-hexane fraction exhibited no significant cytotoxicity (Fig. 2B), whereas weak cytotoxicity was observed at high concentrations in subfractions MR1–7 of the 85% methanol fraction, except for MR1 and MR5.

**Fig. 3:**
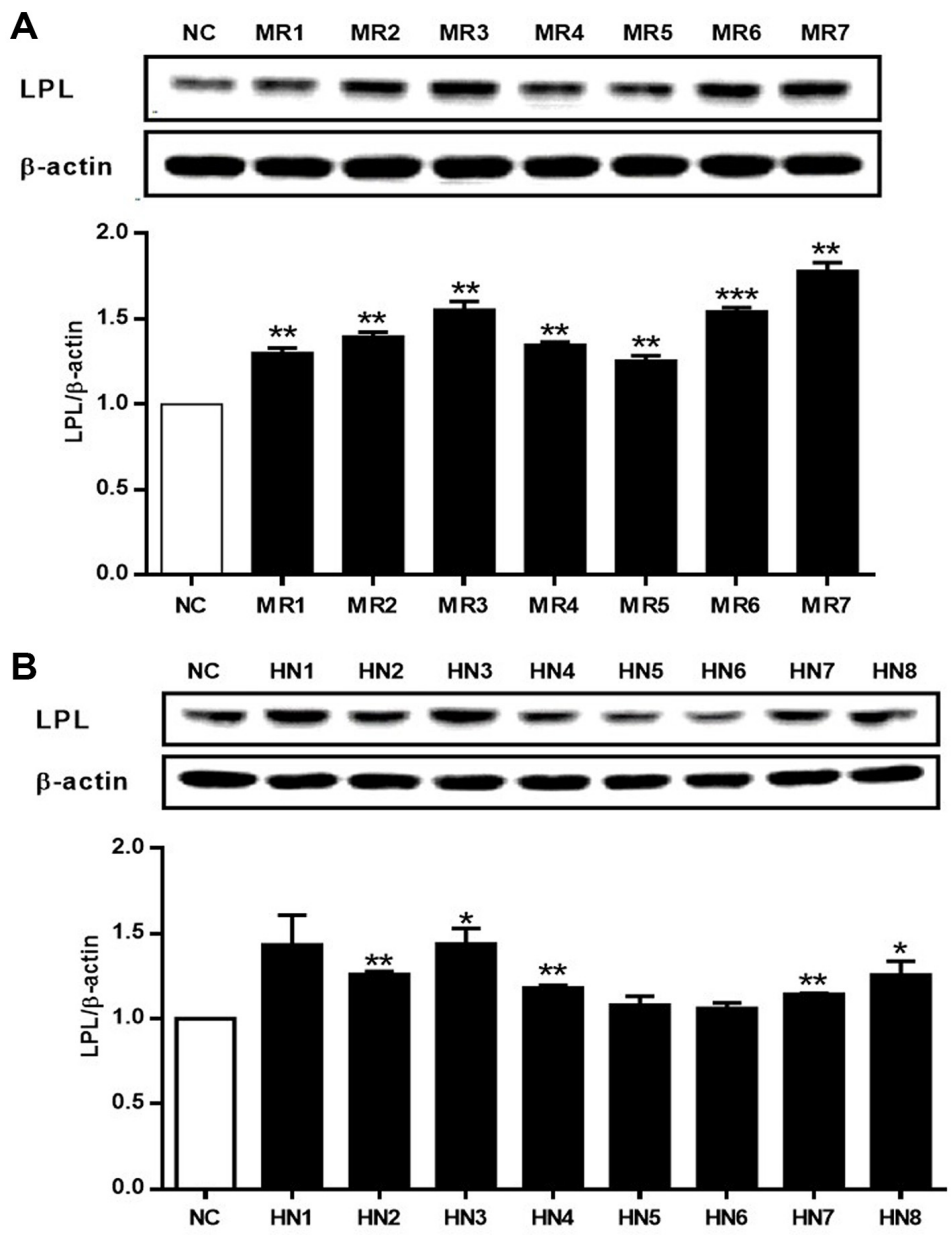
The effects of subfractions of *Oxyrrhis marina* extract on LPL expression in HepG2 cells. HepG2 cells were treated with subfractions MR1–7 of the 85% methanol fraction and subfractions HN1–8 of the *n*-hexane fraction of *O. marina* extract. After 24 h incubation, the protein level of LPL was quantified using the western blot assay. Results are presented as the mean ± SD of triplicate experiments; *p < 0.05, **p < 0.01, and ***p < 0.001 compared to non-treated cells. NC: non-treated control, LPL: lipoprotein lipase.

**Effects of *Oxyrrhis* polar fractions on lipase activity.** Based on the cytotoxicity results, the effects of the fractions of *O. marina* on LPL expression in HepG2 cells were evaluated. Cells were treated with fractions at a concentration of 20 μg/ml, and the protein level of LPL was quantified via western blot analysis after 24 h. As shown in Fig. 3, the 85% methanol fraction and its subfractions generally increased LPL protein expression in HepG2 cells, with MR3 and MR8 exhibiting the most prominent activity. Similarly, treatment with the *n*-hexane fraction and its subfractions also enhanced LPL protein expression, except for HN5 and HN6. In particular, MR3 and MR8 of the 85% MeOH extract demonstrated the highest activity, increasing LPL expression by 1.5- to 2-fold compared to the untreated control group (NC).

### Improvement in Blood Circulation by *Oxyrrhis* Fractions

**Fig. 4:**
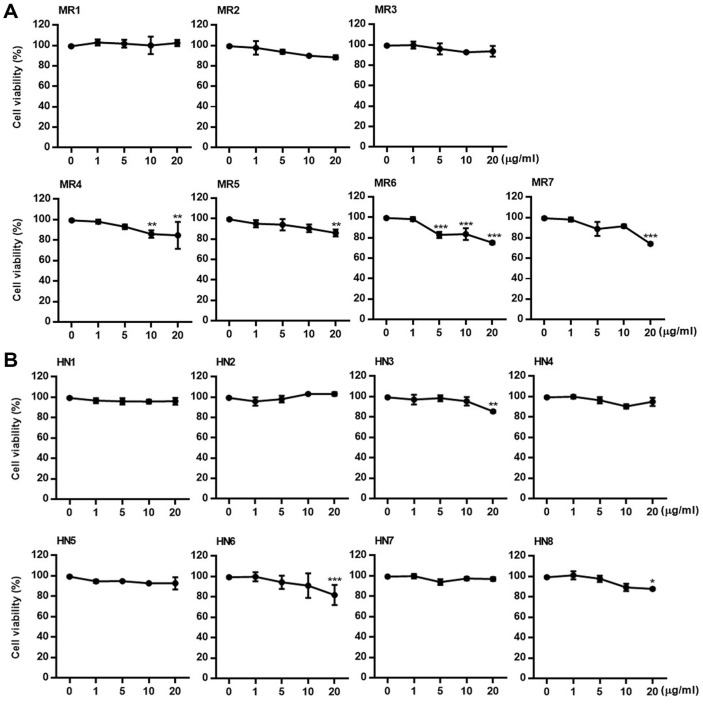
Cytotoxicity of subfractions of *Oxyrrhis marina* extract against HUVECs. HUVECs were treated with subfractions MR1–7 of the 85% methanol fraction and subfractions HN1–8 of the *n*-hexane fraction of *O. marina* extract. After 48 h incubation, cell viability was measured using the MTT assay. Results are presented as the mean ± SD of triplicate experiments; **p < 0.01 and ***p < 0.001 compared to non-treated cells.

**Cytotoxicity of *Oxyrrhis* polar fractions.** The cytotoxicity of the fractions of *O. marina* against HUVECs was evaluated using the MTT assay. HUVECs were treated with fractions at concentrations of 1, 5, 10, and 20 μg/ml. The MTT assay was performed after 48 h of incubation. Similar to the cytotoxicity results observed in HepG2 cells, subfractions of the 85% methanol fraction (except for MR1-3) exhibited mild cytotoxicity in HUVECs, whereas subfractions HN1-8 of the *n*-hexane fraction generally showed no significant cytotoxicity (Fig. 4).

**Fig. 5:**
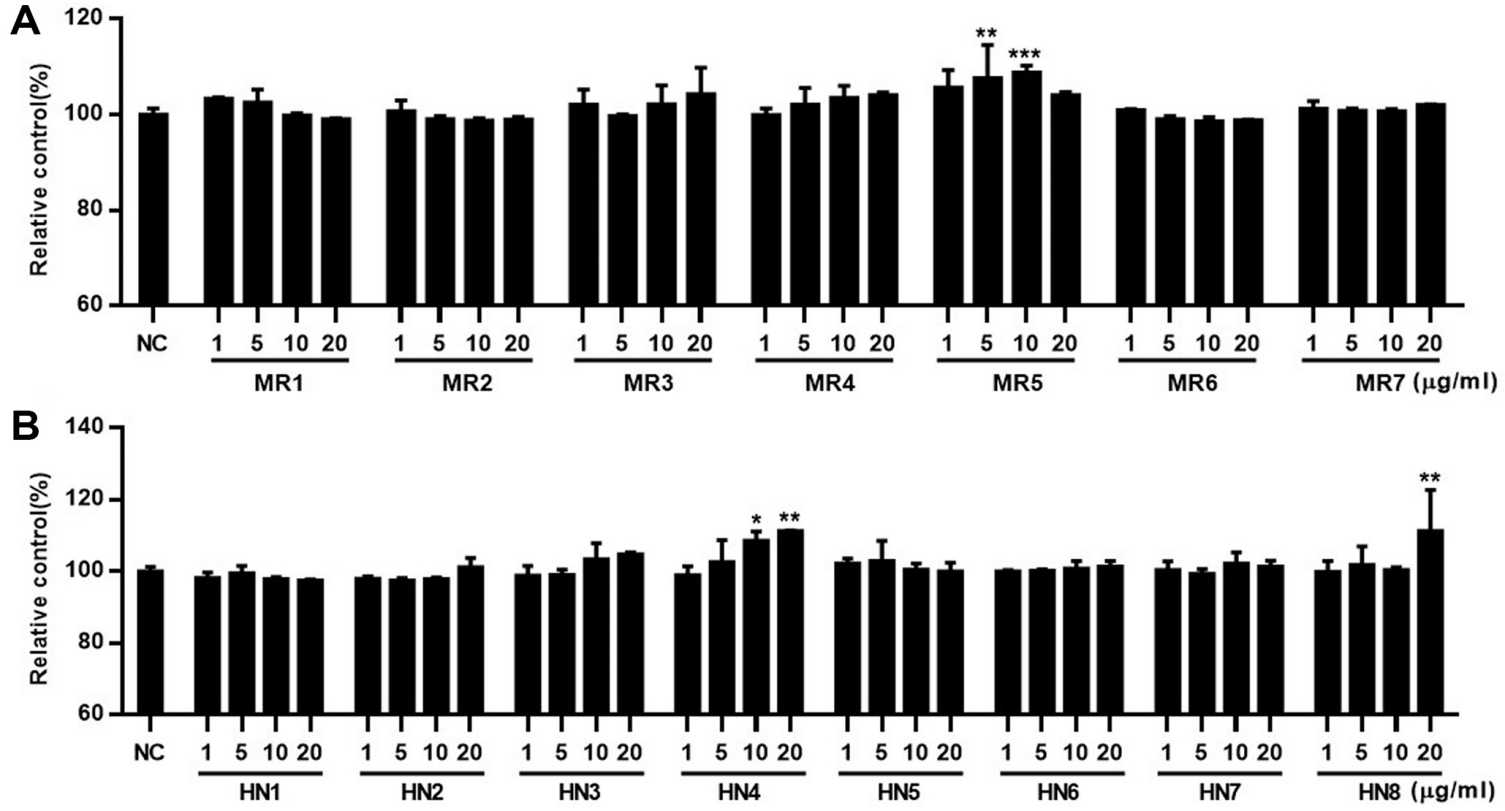
The effects of subfractions of *Oxyrrhis marina* extract on the nitric oxide (NO) production in HUVECs. HUVECs were treated with subfractions MR1–7 of the 85% methanol fraction and subfractions HN1–8 of the *n*-hexane fraction of *O. marina* extract. After 48 h incubation, the NO content was measured using the Griess assay. Results are presented as the mean ± SD of triplicate experiments; *p < 0.05, **p < 0.01, and ***p < 0.001 compared to non-treated cells. NC non-treated control.

**Effects of *Oxyrrhis* polar fraction on NO secretion.** The effects of *O. marina* fractions on nitrite levels were evaluated in HUVECs using the Griess assay. Cells were treated with the fractions at a concentration of 20 μg/ml for 48 h, and the nitrite released into the culture medium was measured. Among the subfractions MR1-MR7 of the 85% methanol fraction, MR5 showed significant nitrite production-enhancing activity at concentrations of 5-10 μg/ml. In the subfractions of the *n*-hexane fraction, HN4 and HN8 exhibited notable activity. In particular, HN4 demonstrated a concentration-dependent increase in nitrite production over the range of 1-20 μg/ml (Fig. 5).

## Discussion

CVDs, which are characterized by complex pathophysiologies, are a leading cause of morbidity and mortality worldwide. A significant contributor to CVD development is the dysregulation of triglyceride metabolism and impaired endothelial function, both of which are crucial in maintaining cardiovascular health. In this study, we explored the potential of *O. marina*-derived extracts, specifically focusing on their effects on LPL expression and NO production, both of which are key physiological players in cardiovascular health.

LPL is an essential enzyme involved in the hydrolysis of neutral fats, particularly triglycerides, into free fatty acids and glycerol. LPL activity is primarily localized in the endothelial cells of various tissues, such as adipose tissue, skeletal muscle, and the heart, where it plays a pivotal role in triglyceride clearance and lipid metabolism. Previous studies have shown that increased LPL activity is associated with improved lipid profiles, particularly the reduction of triglycerides in circulation, which in turn can reduce the risk of atherosclerosis and other CVDs [18, 19]. Our results show that subfractions from the 85% methanol and *n*-hexane extracts of *O. marina* significantly increased LPL expression in HepG2 cells, suggesting that these extracts may facilitate triglyceride metabolism and potentially improve lipid profiles. This aligns with previous findings where marine-derived compounds, such as EPA and DHA, have been shown to enhance LPL activity and reduce triglyceride levels, supporting the potential of *O. marina* extracts as functional ingredients for managing CVD risk [20]. In addition to its role in lipid metabolism, NO is a critical molecule for vascular health, playing an essential role in endothelial function. NO is synthesized by eNOS and is a potent vasodilator, helping to regulate blood pressure and maintain blood flow by relaxing smooth muscle cells in the vasculature [21]. Impaired NO production is closely linked to endothelial dysfunction and hypertension, two common risk factors for cardiovascular diseases. Our study demonstrates that *O. marina* fractions, particularly MR5 from the 85% methanol extract and HN4 from the *n*-hexane fraction, significantly enhanced NO production in HUVECs, suggesting that these fractions may contribute to improved blood circulation. This finding is consistent with previous studies, which have reported that marine-derived oils, rich in polyunsaturated fatty acids like EPA and DHA, enhance NO bioavailability and promote vascular health [22, 23].

To further understand the molecular mechanisms underlying these effects, additional analyses on the signaling pathways involved were considered. Studies have indicated that LPL expression is regulated by peroxisome proliferator-activated receptors (PPARs), particularly PPAR-γ, which plays a crucial role in lipid metabolism and adipogenesis. The activation of PPAR-γ has been shown to enhance LPL expression, facilitating triglyceride clearance and lipid homeostasis. Given that certain marine-derived compounds, such as omega-3 fatty acids, act as PPAR agonists, it is plausible that bioactive constituents in *O. marina* may exert their effects through similar pathways. Further investigation into the activation of PPAR-γ and other transcription factors, such as sterol regulatory element-binding proteins (SREBPs), will be essential to elucidate the precise molecular interactions.

The ability of *O. marina* extracts to modulate both LPL expression and NO production indicates their potential as multifunctional agents for promoting cardiovascular health. The positive effects observed on LPL activity and NO production suggest that these extracts could help manage both lipid metabolism and endothelial function, two critical factors in the prevention and treatment of cardiovascular diseases. The concentration-dependent enhancement of nitrite production in the Griess assay further supports the hypothesis that the extract or fraction obtained *O. marina* may improve vascular tone and circulation, which are vital in managing hypertension and reducing CVD risk. It is noteworthy that the study findings also point to the selective activity of different subfractions, with some showing stronger effects on either LPL expression or NO production. For example, MR5 and HN4 exhibited the most potent effects on NO production, whereas MR3 and MR8 demonstrated the highest LPL-enhancing activity. These differences in bioactivity suggest that *O. marina* extracts contain a diverse range of bioactive compounds that may act synergistically to provide cardiovascular benefits.

This study supports the potential of *O. marina*-derived extracts as effective agents in improving cardiovascular health by modulating key biomarkers such as LPL and NO. The results contribute to a growing body of evidence advocating for the use of marine-derived bioactive compounds in the prevention and management of cardiovascular diseases. Future research should focus on identifying the specific active constituents within *O. marina* extracts that are responsible for these effects and determining their precise mechanisms of action at the molecular level. Additionally, clinical trials will be necessary to confirm the efficacy of these extracts in human populations and assess their potential therapeutic applications.

## Conclusion

In this study, we used the human liver cancer cell line HepG2 to screen the effects of *Oxyrrhis* extract on cholesterol synthesis. Similarly, we used HUVECs to measure NO production, a biomarker of vascular health, which is critical for understanding the potential blood pressure-lowering effects of the extracts. In summary, utilizing biomarkers in cell-based experiments is a vital step in the drug discovery pipeline, as it allows for efficient and early screening of potential therapeutic agents. By providing mechanistic insights and confirming bioactivity, this approach ensures that only the most promising candidates will proceed to animal studies and clinical trials, ultimately improving the efficiency and success of biomedical research.
